# Aichi Virus Strains in Children with Gastroenteritis, China

**DOI:** 10.3201/eid1510.090522

**Published:** 2009-10

**Authors:** Shixing Yang, Wen Zhang, Quan Shen, Zhibiao Yang, Jianguo Zhu, Li Cui, Xiuguo Hua

**Affiliations:** Shanghai Jiao Tong University, Shanghai, People’s Republic of China (S. Yang, Q. Shen, Z. Yang, J. Zhu, Li Cui, X. Hua); Jiangsu University, Zhenjiang, People’s Republic of China (W. Zhang); 1 These authors contributed equally to this article.

**Keywords:** Aichi virus, complete genome, phylogenetic analysis, viruses, letter

**To the Editor:** Aichi virus, a member of the *Kobuvirus* genus of the *Picornaviridae* family (*1*,*2*), is a positive-sense, single-stranded RNA virus with a genome of 8,280 nt and a poly(A) tail. The single, large open-reading frame (ORF) encodes a polyprotein of 2,432 aa that is cleaved into the typical picornavirus structural proteins (VP0, VP3, VP1) and nonstructural proteins (2A, 2B, 2C, 3A, 3B, 3C, 3D) (*2*,*3*). Based on the phylogenetic analysis of 519-bp sequences at the 3C–3D (3 CD) junction, Aichi viruses can be divided into 2 genotypes, A and B, with ≈90% sequence homology (*4*).

Little is known about the epidemiology of Aichi virus. Its presence in fecal specimens of children having diarrhea has been demonstrated in several Asian countries (*5*,*6*), Brazil and Germany (*7*), France (*8*), and Tunisia (*9*). Some reports showed a high level of seroprevalence in adults (*7*,*10*), which suggests widespread exposure to Aichi virus during childhood.

In the present study, 445 fecal samples were collected during April 2008–March 2009 from children 0 to 6 years of age who were hospitalized with acute diarrhea in Shanghai Children’s Hospital, People’s Republic of China. Ninety-two 2–5-year-old children from 3 childcare centers in Shanghai City were included as healthy control subjects. Viral nucleic acid was extracted from 10% stool suspensions in phosphate-buffered saline (pH 7.5) by QIAamp Viral RNA kit (QIAGEN, Hilden, Germany), according to the manufacturer’s instructions. Screening for Aichi viruses was done by reverse transcription–PCR (RT-PCR) with the primers described by Yamashita et al. (*4*), by using a Takara OneStep RT-PCR kit (TaKaRa, Dalian City, Japan). RT-PCR–amplified DNA fragments of the expected sizes, as determined by agarose gel electrophoresis, were excised from the gel, extracted, purified, and sequenced in a 3730 DNA Analyzer (Applied Biosystems, Foster City, CA, USA). Sequence alignment and phylogenetic analysis were performed by using the ClustalX (http://bips.u-strasbg.fr/fr/Documentation/ClustalX) and MEGA4 software (www.megasoftware.net), respectively. Aichi virus RNA was detected in 8 samples (1.8% incidence). The PCR-amplified products of 8 strains were sequenced, and the resulting sequences were submitted to GenBank with the strain names Chshc1–8 and accession nos. FJ890516–FJ890523. Sequence analysis, based on the 529-bp sequences, showed that the isolates shared 98.2%–99.6% identities with each other, which suggests that they can be considered a unique strain. When compared with all Aichi virus strains available in GenBank, the 8 sequences shared 91.3%–96.9% sequence identities, except for a strain from France, DQ145759, which had only 87.2% sequence homology with the other strains in this study. Phylogenetic analysis of those Aichi virus strains, based on the 519-bp sequence, showed that the 8 strains belonged to genotype B (Figure, panel A) and closely clustered with a Japanese strain AB092830, sharing 96.9% sequence identity with it. The 8 Aichi virus–positive samples were further investigated for norovirus, sapovirus, rotavirus, astrovirus, and adenovirus types 40 and 41 by RT-PCR with the primers described (*9*). Results indicated that one of the samples was also positive for astrovirus, of which the 348-bp–specific fragment was sequenced and deposited in GenBank (accession no. GQ292771). No Aichi virus was detected in samples from the 92 healthy control subjects.

**Figure:**
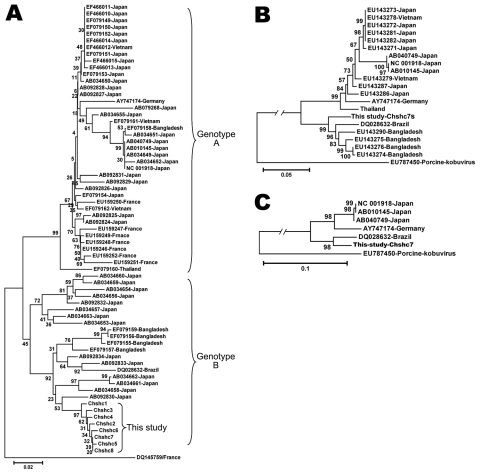
Phylogenetic tree constructed by using the neighbor-joining method and evaluated using the interior branch test method with MEGA4 software (www.megasoftware.net). Percentage of bootstrap support is indicated at each node. GenBank accession number, source, and country of origin are indicated. A) Phylogenetic tree constructed based on the 519-nt segment in the 3C/D junction region, a genotype C Aichi virus strain is included as an outgroup. Phylogenetic trees constructed from the capsid gene (B) and complete genome of Aichi virus (C); the porcine kobuvirus is included as an outgroup. Scale bars indicate nucleotide substitutions per site.

The complete genomic sequence of strain Chshc7 was then determined by using 13 sets of specific oligonucleotide primers designed on the complete genome of 4 Aichi virus strains (NC_001918, AB010145, DQ028632, AY747174). Results showed that the full genome of this virus strain was 8,244 nt and contained a ORF frame with a length of 7,299 nt, encoding a putative polyprotein precursor of 2,433 aa. This ORF is preceded by a 5′ untranslated region (UTR) at least 712 nt in length. The 3′ UTR measure 237 nt, excluding the poly(A) tract. Base compositions of the strain were found to be A, 19.8%, C, 37.8%, G, 20.9%, and U, 21.5%. The polyprotein precursors of this Aichi strain comprise a predicted leader protein of 170 aa and putative VP0, VP3, and VP1 proteins with lengths of 370 aa, 224 aa, and 278 aa, respectively. Regarding the nonstructural proteins, lengths of 111 aa, 165 aa, and 335 aa are predicted for 2A, 2B, and 2C, and of 93 aa (29 aa, 190 aa, 468 aa) for 3A (3B, 3C, 3D).

Phylogenetic trees were constructed on the basis of the capsid protein gene (Figure, panel B), complete genome sequences of the strain Chshc7 (Figure, panel C), and those sequences available in GenBank. Both phylogenetic trees indicated that the strain Chsh7 closely clustered with the Brazilian strain DQ028632, which confirmed that Chsh7 belonged to genotype B. Sequence alignment showed that Chsh7 and the Brazilian strain (DQ028632) shared 95.3% and 98.1% sequence identities with each other over the complete genome and putative amino acid sequences, respectively, which suggested that the 2 strains might come from a common ancestor. These results will provide useful information for further epidemiologic study of Aichi virus in China.
